# Women attending antenatal care as a sentinel surveillance population for malaria in Geita region, Tanzania: feasibility and acceptability to women and providers

**DOI:** 10.1186/s12936-023-04480-y

**Published:** 2023-02-24

**Authors:** Courtney Emerson, Stephen Ulimboka, Ruth Lemwayi, Alen Kinyina, Samwel L. Nhiga, Sijenunu Aaron, Japhet Simeo, Chonge Kitojo, Erik J. Reaves, Mary Drake, Yahaya Hussein, Leila Bungire, Julie R. Gutman, Peter J. Winch

**Affiliations:** 1 Malaria Branch, U.S. President’s Malaria Initiative, U.S. Centers for Disease Control and Prevention, Atlanta, GA USA; 2 PMI Boresha Afya, Jhpiego, Dar Es Salaam, Tanzania; 3 National Malaria Control Programme, Ministry of Health, Dodoma, Tanzania; 4 Regional Health Management Team, Geita, Geita Region Tanzania; 5 U.S. President’s Malaria Initiative, U.S. Agency for International Development, Dar Es Salaam, Tanzania; 6 U.S. President’s Malaria Initiative, U.S. Centers for Disease Control and Prevention, Dar Es Salaam, Tanzania; 7 Department of Health, Nutrition and Social Welfare, President’s Office Regional Administration and Local Government, Dodoma, Tanzania; 8 Malaria Branch, Division of Parasitic Diseases and Malaria, U.S. Centers for Disease Control and Prevention, Atlanta, GA USA; 9 Department of International Health, Johns Hopkins Bloomberg School of Public Health, Baltimore, MD USA; 10 Department of Reproductive, Maternal, and Child Health, Ministry of Health, Dodoma, Tanzania

**Keywords:** Malaria, Pregnancy, Antenatal care, Surveillance, Tanzania

## Abstract

**Background:**

Measurement of malaria prevalence is conventionally estimated through infrequent cross-sectional household surveys that do not provide continuous information regarding malaria parasitaemia. Recent studies have suggested that malaria parasitaemia prevalence among women attending antenatal care (ANC) correlates with prevalence among children under 5 years old and that pregnant women could be a sentinel population for tracking malaria prevalence. In mainland Tanzania, 97% of women are tested for malaria parasitaemia during first ANC visits. However, acceptability among pregnant women and healthcare providers of collecting malaria risk factor data during ANC visits is limited.

**Methods:**

A tablet-based questionnaire including 15 questions on insecticide-treated net ownership and use and care-seeking for febrile children was introduced at 40 healthcare facilities in Geita Region, Tanzania. Facilities were randomly selected from among those with 15–120 first ANC visits per month. To assess perspectives regarding introduction of the questionnaire, 21 semi-structured interviews were held with providers and facility in-charges at 12 facilities. Thirty pregnant and recently delivered women participated in focus group discussions at seven facilities to assess the acceptability of spending additional time answering questions about malaria risk.

**Results:**

All pregnant women reported that introduction of ANC surveillance and spending 10 more minutes with providers answering questions about their health would be neutral or beneficial. They perceived being asked about their health as standard of care. Providers and in-charges reported that introduction of ANC surveillance was within their scope of practice. Nine of 21 indicated it could potentially benefit women’s health. Six providers expressed concern about staffing shortages and need for reimbursement for extra time and noted that data management occurs after hours.

**Conclusions:**

Pregnant women and providers generally perceived ANC surveillance for malaria as acceptable and positive. Pregnant and recently delivered women saw this as a reasonable and even helpful intervention. To be seen as a part of standard practice, efforts are needed to ensure providers perceive a benefit for ANC clients and that staffing concerns are addressed. In addition, staff should receive feedback related to data submissions regarding malaria prevalence and risk factors among women at their facility, with actions to take.

## Background

Malaria burden remains high in many areas of Tanzania, with 93% of the population living in areas with high malaria risk [1], including Geita Region, in the highly endemic Lake Zone in Northwestern Tanzania. In 2017, the malaria prevalence in children in Geita Region was 17%, while malaria prevalence among pregnant women was 12% [2]. Estimates of malaria burden and intervention uptake in Africa are primarily based upon nationally representative household surveys [3]. However, their expense and infrequency, as well as the fact that they are powered for regional level estimates, limit their utility for timely analysis for operational action by malaria programmes. In recent years, the idea that women attending antenatal care could be used for surveillance of trends in both malaria parasitaemia prevalence and the coverage of malaria control interventions has gained traction. Several studies, including in Tanzania, have demonstrated the correlation between malaria parasitaemia prevalence among pregnant women and prevalence among children under five, a group often targeted for national household surveys [4–7]. The concept of antenatal care (ANC) surveillance for monitoring the prevalence of infectious diseases in the community is not new and was historically a component of surveillance and service delivery for both human immunodeficiency virus (HIV) [8, 9] and sexually transmitted infections such as syphilis [10].

In 2014, Tanzania implemented malaria testing, generally by rapid diagnostic test (RDT), as standard of care at first antenatal care visit (ANC1) as part of the surveillance system [11, 12]; ANC malaria test results are recorded in the Tanzanian National Health Management Information System (HMIS) utilizing District Health Information Software 2 (DHIS2) [13]. These data are triangulated with other routine data to develop malaria transmission strata and determine when and where specific interventions are implemented [14]. It has been hypothesized that in addition to malaria prevalence, a short questionnaire administered to women attending ANC1 could provide useful data on the coverage of malaria control interventions and be used to monitor trends over time; however, the validity compared to the routine population requires systematic assessment. A study was recently conducted including 40 healthcare facilities in Geita Region, Tanzania to assess whether malaria surveillance data (parasitaemia prevalence, population coverage of insecticide-treated nets (ITNs), and care-seeking behaviour for febrile children) collected from ANC correlated with data from routine household surveys [15].

Although there are data demonstrating the validity of ANC surveillance for estimating malaria prevalence, there are limited data on the perspectives of providers, as well as pregnant or recently delivered women themselves; further, none of these studies considered aspects other than surveillance for malaria prevalence [16]. As there is consideration of the role that ANC surveillance could play in monitoring malaria burden and coverage of malaria control interventions, it is critical to hear perspectives from those who would perform the work and receive services [17]. Not taking their perspectives into account can result in reduced program effectiveness [18]. Further, provider attitudes are also important in women feeling cared for and continuing to seek prenatal care [19]. The purpose of this qualitative study is to examine the acceptability to pregnant women and ANC providers of including additional questions during ANC visits to assess coverage of malaria control interventions, and potential use of data by providers.

## Methods

An ANC surveillance questionnaire was implemented in 40 facilities in Geita Region, Northwestern Tanzania, from March 2020–June 2021. Facilities that had between 20 and 120 ANC1 visits per month and had more than one ANC provider working at a time were randomly selected; these criteria were required for a study of a group antenatal care intervention that was intended to be implemented concomitantly. All consenting pregnant women attending ANC1 at selected facilities were asked a set of questions (approximately 10–15, depending on their responses) regarding their household composition, access and use of ITNs, number of children, and care-seeking behaviour for fever amongst any children under five years of age who had a fever in the preceding two weeks (Appendix 1). These questions were consistent with ones used in cross-sectional studies to evaluate coverage indicators and time for questionnaire completion and number of women interviewed each day was measured. Questionnaires programmed into a CommCare [20] database were administered by routine ANC providers. Deidentified data from tablets were uploaded weekly to a secure server.

In-depth interviews and focus groups were conducted in June 2021. Providers were eligible to participate in in-depth interviews if they were full-time employees working at the ANC. Pregnant women and recently delivered women residing in the facility catchment area were eligible to participate in focus groups. A research assistant was placed at each of seven purposively selected facilities (from among the 40). The research assistants recruited a total of 10 providers and 11 facility in-charges for the in-depth interviews and a total of 30 pregnant and recently delivered women for focus group discussions.

## Data collection

Verbal informed consent was given by providers completing semi-structured, in-depth interviews and by pregnant women participating in focus groups following reading of a consent form by the interviewer. Semi-structured interviews and focus groups were conducted in private areas and by trained interviewers fluent in English and Kiswahili who were not associated with the Ministry of Health. Focus group discussions were limited to 4–5 participants to allow the research team to adhere to standard COVID-19 prevention protocols. In addition, interviewers wore face masks at all times when in contact with participants. Participants were offered refreshments, though not remunerated for participation in this study.

In-depth interviews were conducted in the language of choice of the provider (English or Kiswahili) and focus groups were conducted in Kiswahili. Data were collected through pre-developed interview guides and flexibility was allowed to formulation of questions and probing based on participant responses. All interviews were recorded using digital recorders, and extensive notes were also taken to ensure a record of the interview. Providers were asked for descriptions of care for pregnant women, perception of malaria risk amongst pregnant women, data use, and perspectives regarding potential of introducing ANC surveillance questions into the clinic. Questions to pregnant women in focus groups related to factors encouraging use of intermittent preventive treatment of malaria in pregnancy (IPTp) and willingness to be asked additional questions regarding malaria at the 1st ANC visit (ANC1) (i.e., to inform potential introduction of ANC surveillance for malaria). Interviews were recorded and transcribed into Kiswahili, then subsequently translated into English with Google Translate, followed by checking of the translation for errors. The analysis questionnaires, as well as interview guides were developed in relationship to perceived acceptability in terms of the Sekhon, et al.[21] framework including attitudes, fit with values, burden, as well as time costs to assess the feasibility and acceptability of using the ANC clinic to collect data for malaria surveillance.

### Data analysis

Interviews were coded in two ways. Responses to selected questions such as operational challenges (Table 1) were coded in closed-ended formats and summarized in tables. Themes in transcripts were also identified with a combination of deductive coding based on a priori issues reported in the literature, and inductive coding of respondents’ narratives and other findings in the transcripts. Audio recordings were destroyed after transcription and verification of the transcriptions. Analyses across all transcripts and cover sheets were conducted manually and involved development of descriptive codes, organizing concepts, and identifying themes, their repetitions and variations.

**Table 1:** Providers’ and in-charges’ experience with potential introduction of antenatal care (ANC) surveillance for malaria (N = 21)

	N
Think introduction of ANC surveillance would be challenging	6/21
If noted ANC surveillance as challenging, concerns noted:	
Insufficient staff	4/21
Need for compensation for extra duty	3/21
Patient wait times are already long	2/21
Concern that it will make workday even longer	2/21
Potential patient refusal	1/21
Need for training	1/21

## Results

### Feasibility and acceptability of ANC surveillance: perspectives of pregnant and recently delivered women

All 30 women interviewed were supportive of the introduction of ANC surveillance, which was framed to focus group participants as introduction of a 10-min questionnaire during their ANC1 visit asking about malaria prevention in the household. Receiving counseling and being asked questions were perceived as a normal part of care that they expect at ANC. One woman noted that she was used to coming to ANC at 9am and leaving at 12 pm or 1 pm in the afternoon, so the addition or inclusion of 10 min did not seem consequential. One woman noted that she felt it could improve the health of women to receive additional time with a provider being asked about their health practices.

This is part of the normal health services for a pregnant woman …it is not only normal for me, ask anyone here…you will get the same answer…they will tell you that they are ready for it…so I will be ready.

I will stand to it and hold for that time…for us most often when we go to the health facility for clinics, we normally spend longer time to obtain care, treatment and other services; when we arrive at 9am in the morning, we normally leave at 12 or 1 pm in the afternoon. So, what is 10 min to us? It will just be normal.

### Feasibility and acceptability of ANC surveillance: perspectives of providers

Fifteen of 21 providers thought that introduction of ANC surveillance and the associated questionnaire with women at ANC1 would be both feasible and acceptable. Some felt it had potential to contribute to improved patient care. Others felt that staffing shortages, long hours, and the need to address large numbers of patients would make this difficult and necessitate incentives for providers (Table 1).

#### Feasible and acceptable

Fifteen Providers indicated that it was feasible to spend additional time to ask women attending ANC questions about malaria interventions, citing that by doing so, it would make the services they deliver more productive and valuable to attending women. Moreover, some providers reported this is just part of their responsibilities.

Because I do not know what you are doing, but if it is in the context of the responsibility of helping pregnant women I see it as a normal responsibility like other responsibilities we do because if a pregnant mother needs to be cared for, and to have her baby safe, things must be done all, so I do not consider it necessary to add to my burden, but to help her who needs help. (Even if) we are working outside the clock? We do. -Provider.

#### Potential contribution to quality of patient care

Seven providers explicitly noted that that they would be willing to support ANC surveillance if it supports or influences the care of the pregnant woman.

For my part I do not see it as a big responsibility, because there are things that you cannot jump no matter what you do, you cannot get to the mother, not educate her, and just start caring for her. You will not have treated her fairly, even if at the first attendance you taught her. Just because this is a matter of adding responsibilities, does not mean that this thing takes long, because one mother can sit with you for two minutes and you have finished asking her your questions and you have educated her, then she leaves. -Provider.

#### Perceived implementation challenges: staff shortages and burden to providers

Although 15 providers reported spending additional time on these new questions was feasible and acceptable, staff shortages, patient waiting time, and the need to address sick patients as a priority were identified as limiting factors in 6 interviews. In three interviews, feasibility and acceptability were said to be possible if providers were to receive extra duty allowances.

My response to you is that spending additional time would mean a burden to healthcare providers…the only way to keep the providers motivated and for them to work efficiently and handle these extra duty activities well…they need to be considered with payment of extra duty allowances. -Facility In-Charge.

Let me just say the challenge is that the shortage of staff really makes the job harder. It may reach the point where you have to provide services here, and then again, they are waiting for you. So, it makes it difficult because you can be brought to someone who is sick and not in good condition, where you have to stop. -Facility In-Charge.

#### Perceived implementation challenges: need for technical assistance and to address staff shortages

In some facilities, the addition of a surveillance questionnaire was considered feasible by providers, but they insisted that there should be some training and technical assistance. They also noted the need to address existing staff shortages but communicated that the intervention could benefit the district in better understanding trends.

For us here we welcome the project, but it will be important for them to come up with trainings and guidelines on malaria prevention and treatment as well…another challenge will be how to accommodate all these because we have many clients at the ANC with few staff to serve them. -Facility in-charge.

There are also days when you can find almost everyone, we are all at work, no one is on vacation or having a problem…We are planning how we will make all the work go. -Facility in-charge.

### Utilization of data for informing malaria programme management

With regards to malaria burden, there were mixed opinions; some providers considered the disease a problem in their communities and others had opposing views. Those who viewed malaria as a problem pointed out that malaria is not only a problem to pregnant women but the whole community. They emphasized that many people test positive for malaria, which makes it a chronic problem in their area. Further, most of the providers indicated that pregnant women are at higher risk of having malaria than non-pregnant women.

Most of the facility in-charges interviewed affirmed their involvement in preparation of monthly reports of malaria. Some of them further indicated that monthly meetings are conducted for malaria report review in their facilities. After reviews, the reports are sent to district level. The indicators for malaria that facility in-charges focused on include: the number of pregnant women testing positive for malaria, and the numbers of pregnant women that received IPTp and ITNs in a particular month. With respect to trends in confirmed cases of malaria, most of the facility in-charges mentioned that most cases of malaria occur between October and April.

#### Main areas of focus in data collection

The main areas of focus in facility data collection were on composition of monthly reports and attending to key required indicators.

Of course, we all cooperate to compose the monthly reports, a representative from each unit represents their data and then we compile them as one document to be reviewed by all before sending it to the District Council. -Facility in-charge .

Main indicator is the number pregnant women who test positive for malaria compared to the total number of tested pregnant women. Other indicators include gender role, number of pregnant women who received SP but overall, the indicator which identify prevalence of malaria is a number of malaria positive pregnant women out of all malaria tested pregnant women. -Facility in-charge.

#### Data not used to track pregnant women

Despite the availability of facility ANC records, these data are not used to track changes in malaria prevalence among pregnant women. The reasons given by some of the facility in-charges include limited time and since there is no requirement for reporting the number of pregnant women who test positive for malaria per month at higher levels, the facility does not review these trends. However, the few who review malaria reports affirmed there is a yearly variation in pregnant women testing positive for malaria by RDT.

#### Previous quality improvement initiatives and recognition of limitations in data entry

There were some quality improvement activities and meetings related to malaria data that had been convened previously to which several providers referred. One provider noted that when there are a lot of patients, it is also possible to deliver services and not record the information, such as delivery of IPTp.

I grew up paying close attention to ANC information because I had been the chairman of Quality Improvement and that was among the indicators that we grew up dealing with and that was there. So, within that year we tried to improve it in some places even though we came up with a challenge if the medicine ran out. -Facility In-Charge.

#### Data not a priority or a challenge to collect and maintain

Some providers noted that data were not a priority or were a challenge to collect and maintain.

So now we say that when the workload comes to a point where you may find the service provider tired, I can't deny it, it may happen, because you find yourself tired, and there are a lot of people now and you find yourself giving it to him but not recording it, it is possible. -Provider.

Interviewer: In your monthly reports when you submit to the Council do you just submit the report, or do you make suggestions for the intervention to be done for something?

Response: *We are just writing a report.* -Facility In-Charge.

#### Challenges in malaria commodity stock

Several providers noted challenges regarding malaria commodity stock and a sense from some that although there was a report being submitted, there was not necessarily a feedback loop at the healthcare facility level.

The biggest challenge and the other challenge is this one of mosquito nets but at the moment the nets are sitting in line. There is a period and we stayed for more than 6 months without mosquito nets and insist we are told we have not applied but if you enter the system, we have requested, or you find we are brought a few mosquito nets and they end prematurely but this is not a big gap except for those anti-malarial drugs for pregnant mothers. -Facility In-Charge.

#### Taking action from data

There was no clear information regarding the threshold of malaria cases among pregnant women that would prompt a provider to explore the causes of increased positivity rates. However, most of the facility in-charges indicated that if they should observe an increased number of pregnant women testing positive, their first action would be to assess the cause of the increase and design an intervention to address the problem.

First, I will have to assess the cause of that huge increase in malaria, communicate with community health workers to find what is the cause. Then we will make a plan for intervention of which health education on preventative measures of malaria will be provided to the communities at household level. Also, another action to be taken is environment cleanliness to eliminate mosquito breeding areas and supply mosquito nets especially to pregnant women if possible and teach them importance of using nets. -Facility In-Charge.

## Discussion

Across sub–Saharan Africa, a high proportion of women attend ANC at least once, thus data collection during ANC may be a good option for surveillance of malaria parasitaemia and coverage of malaria control interventions. ANC surveillance was acceptable to women, who felt that it was within standard of care provided at ANC. While providers and facility in-charges reported being willing to spend time on ANC surveillance if it contributed to patient care, several mentioned the already heavy workload and the additional work as possible barriers to implementation.

In implementing an intervention that requires additional time from women and providers, it is important to ensure that implementing the intervention will not adversely affect the uptake or quality of care. In general, women were not opposed to answering questions about their household ownership and usage of ITNs and care seeking behaviours; the additional time it took to answer questions (which averaged 4.7 min) was seen as negligible compared to the time that they spent waiting for services. Responses to the questions regarding ITNs and care seeking used in this, or a similar ANC surveillance questionnaire could inform education to support positive health actions for women and their families (Appendix 1). This could have a beneficial impact on ANC attendance, if women perceive this as improving the quality of care, and if they feel these questions demonstrate that the providers care about them. In other studies, exploring the barriers to ANC attendance, women frequently report that providers are rude [22, 23] and not empathetic [24, 25]. Thus, if a woman perceives that the provider is interested in her wellbeing, this might encourage her to attend additional ANC.

Implementation of the questionnaire is likely to burden healthcare providers to a greater extent than patients. Implementation of testing for malaria with an RDT is already underway in all ANC facilities in Tanzania. Because blood for the RDT is obtained at the same time as blood for other routine labs, performing the RDT led to minimal additional workload for the providers, as documented in previous studies [16, 26–28]. On the other hand, providers noted that implementation of the questionnaire would add to their workload and cited a need for training as has been found in other studies [28]. While some felt it would be worthwhile to ask these questions if they improved the care that women received, others felt that there would be no benefit to these questions.

Providers commented that addressing urgent health conditions of women on arrival is a top priority. The idea that data-focused work is something that happens at the end of the day rather than during the day is challenging with ANC surveillance as questionnaire responses would need to be collected when pregnant women are present. These data collection forms must be as short as possible. Some providers interviewed noted working long days to complete duties and concerns about patient wait times have been documented in other studies when additional duties were added to provider tasks [29]. A 10-min form could be challenging in these real-world conditions. Providers interviewed a median of seven women per day (interquartile range 4–14); this would add on average one hour (and up to two hours) to provider workloads. There is already a nationwide system for collection of data regarding malaria testing at ANC. Implementing a sampling frame, or set cadence of quarterly or semi-annual data collection, to ask these questions of a limited number of women could be accomplished with tablet-based data collection and would overall reduce the associated workload. Additionally, because a minimum number of responses are needed to generate reliable data, it likely does not make sense to implement this ANC surveillance strategy at very low volume ANC clinics. It may therefore make sense to implement in higher volume clinics and assign a dedicated person to collect the data. It should be noted that because this was implemented in the context of a study, verbal consent was obtained from all women prior to initiating data collection. This added time to the study workload but would not be required if implemented routinely by health facilities. In terms of data use, there was some attention to quality improvement, but there did not seem to be a large sense of using the malaria data from the questionnaire for specific actions at the facility level. There is thus a need to define how this data could be used and at what level. Malaria surveillance among pregnant women has been proposed as a component of the strategy to develop a multilayered stratification [14] and support Tanzania’s move towards tailoring interventions at the council level [30].

In terms of limitations, the investigators spoke with a relatively small number of pregnant women and providers; thus, it may not represent the perspectives of individuals nationwide. In addition, the size of focus groups was limited due to COVID-19 precautions.

It will be important to better understand any operational issues related to ANC surveillance for malaria, as well as the perspectives of providers and women regarding the extent to which the questionnaire data collection activity contributes to clinical care (in addition to the benefit of malaria testing amongst pregnant women). It will also be important to determine how data collection and analysis would flow with the addition of questions asked along with the malaria test conducted at ANC.

## Conclusions

Establishment of pregnant women as a sentinel surveillance population for malaria in Tanzania was acceptable to pregnant women. However, acceptability among providers, and healthcare facility in-charges, would in many cases be incumbent upon careful consideration of workload and potential staffing increases or reimbursement. To be scaled more broadly, there would need to be a clear plan for data utilization, at regional and national levels, in addition to facility levels. In many facilities, data utilization remains low. Most scientific literature has identified ANC surveillance as an adjunct to existing surveillance and survey methodologies. If it has the potential to replace other data collection methodologies it would be important to explore additional data collection support at the facility level so as not to be an additional burden for both ANC attendees and providers.

## Data Availability

Data from this study can be accessed upon request through the corresponding author who will submit a formal request to the Ministry of Health. Data have been made available on the USAID Development Data Library (DDL; https://data.usaid.gov/) as restricted access pending approval from Tanzania government. Data can also be requested from Jhpiego by writing to opendatahelp@jhpiego.org under a data use agreement.

## Malaria ANC surveillance questionnaire for use at 1st ANC visit

**Table:**

#	Question	Response	Skip pattern
	How many people usually live in your household?	Number of people |____|_____|	
	How many insecticide treated mosquito nets (LLIN) does your household have?	Number of nets |____|_____|	If "0" then skip to 7
	Did you sleep under an LLIN last night?	YES…………………………1 NO…………………………0.2	
	How many children under 5 slept in your household last night?	Number of children |____|_____|	If "0" then skip to 7
	How many of those children slept under a net last night?	Number of children |____|_____|	
	Is this your first pregnancy?	YES…………………………1 NO…………………………0.2	If YES then END
	How many prior pregnancies have you had?	Number of pregnancies |____|_____|	
	How many children under 5 do you have?	NUMBER OF CHILDREN |____|____|	If "0" then END
	Have any of your children who are under 5 years old been ill with a fever at any time in the last 2 weeks?	YES…………………………1 NO…………………………0.2	If NO then END
	For each child aged < 5 years with fever in the past 2 weeks: at any time during the illness, did you seek any advice or treatment for the illness from any source?	YES…………………………1 NO…………………………0.2	If NO then END
	Where did you seek treatment?	GOVERNMENT HOSPITAL……………………01 GOVERNMENT HEALTH CENTER…………0.02 GOVERNMENT HEALTH POST………………03 MOBILE CLINIC ……………………………………04 FIELDWORKER ……………………………………05 OTHER PUBLIC SECTOR………………………0.06 PRIVATE HOSPITAL/CLINIC…………………0.07 PHARMACY ……………………………………….0.08 PRIVATE DOCTOR ………………………………09 MOBILE CLINIC ………………………………….0.10 FIELDWORKER ……………………………………11 OTHER PRIVATE MEDICAL SECTOR…….0.12 SHOP …………………………………………………13 TRADITIONAL PRACTITIONER ……………14 MARKET ……………………………………………15 ITINERANT DRUG SELLER……………………16 OTHER……………………………………………………97	
	How many days after the illness began did you first seek advice or treatment for this child?	NUMBER OF DAYS	
	Did this child have blood taken from his/ her finger or heel for malaria testing?	YES…………………………1 NO…………………………0.2	
	At any time during the illness, did this child take any drugs for the illness?	YES…………………………1 NO…………………………0.2	Continue END
	What drugs did this child take?	ANTIMALARIAL DRUGS ORAL ARTEMISININ COMBINATION THERAPY (ACT). …………………………………01 SP/FANSIDAR ……………………………………02 CHLOROQUINE……………………………………03 AMODIAQUINE……………………………………04 QUININE…………………………………………….0.05 OTHER (specify) ___________________06 INJECTION/RECTAL ARTESUNATE INJECTION…………………0.07 QUININE INJECTION…………………………08 RECTAL ARTESUNATE………………………09 ANTIBIOTIC DRUGS PILL/SYRUP …………………………….0.10 INJECTION/IV……………………………11 DON'T KNOW…………………………………………12	

## References

[1] WHO (2021). World malaria report.

[2] Ministry of Health Community Development, Gender, Elderly and Children Tanzania Mainland], Ministry of Health [Zanzibar], National Bureau of Statistics (NBS), Office of the Chief, Government Statistician (OCGS), ICF. Tanzania Malaria Indicator Survey 2017. Dar es Salaam, Tanzania and Rockville, USA; 2017.

[3] DHS Program. Malaria Indicator Surveys 2022 https://dhsprogram.com/methodology/survey-types/mis.cfm.

[4] van Eijk AM, Hill J, Noor AM, Snow RW, ter Kuile FO (2015). Prevalence of malaria infection in pregnant women compared with children for tracking malaria transmission in sub-Saharan Africa: a systematic review and meta-analysis. Lancet Glob Health.

[5] Kitojo C, Gutman JR, Chacky F, Kigadye E, Mkude S, Mandike R (2019). Estimating malaria burden among pregnant women using data from antenatal care centres in Tanzania: a population-based study. Lancet Glob Health.

[6] Hellewell J, Walker P, Ghani A, Rao B, Churcher TS (2018). Using ante-natal clinic prevalence data to monitor temporal changes in malaria incidence in a humanitarian setting in the Democratic Republic of Congo. Malar J.

[7] Brunner NC, Chacky F, Mandike R, Mohamed A, Runge M, Thawer SG (2019). The potential of pregnant women as a sentinel population for malaria surveillance. Malar J.

[8] Dee J, Garcia Calleja JM, Marsh K, Zaidi I, Murrill C, Swaminathan M (2017). HIV surveillance among pregnant women attending antenatal clinics: evolution and current direction. JMIR Public Health Surveill.

[9] Bakari JP, McKenna S, Myrick A, Mwinga K, Bhat GJ, Allen S (2006). Rapid voluntary testing and counseling for HIV: acceptability and feasibility in zambian antenatal care clinics. Ann N Y Acad Sci.

[10] Korenromp EL, Mahiané SG, Nagelkerke N, Taylor MM, Williams R, Chico RM (2018). Syphilis prevalence trends in adult women in 132 countries—estimations using the Spectrum Sexually Transmitted Infections model. Sci Rep.

[11] Ministry of Health Community Development, Gender, Elderly and Children Tanzania. National Malaria Strategic Plan 2015–2020 Dar es Salaam, Tanzania, 2015.

[12] Ministry of Health Community Development, Gender, Elderly and Children Tanzania. Antenatal Care Guidelines. Dar es Salaam, Tanzania, 2018.

[13] Willilo RA, Molteni F, Mandike R, Mugalura FE, Mutafungwa A, Thadeo A (2016). Pregnant women and infants as sentinel populations to monitor prevalence of malaria: results of pilot study in Lake Zone of Tanzania. Malar J.

[14] Thawer SG, Chacky F, Runge M, Reaves E, Mandike R, Lazaro S (2020). Sub-national stratification of malaria risk in mainland Tanzania: a simplified assembly of survey and routine data. Malar J.

[15] Nhiga SL, Kinyina A, Assenga M, Munsey A, Lash R, Almeida A (2021). Assessing the utility of antenatal care surveillance in Tanzania for monitoring coverage of malaria control interventions. Am Soc Trop Med Hyg..

[16] Kitojo C, Chacky F, Kigadye ES, Mugasa JP, Lusasi A, Mohamed A (2021). Acceptability of single screening and treatment policy for the control of malaria in pregnancy: perceptions of providers and pregnant women from selected health facilities in Lindi region. Tanzania Malar J.

[17] Mutale W, Chintu N, Amoroso C, Awoonor-Williams K, Phillips J, Baynes C (2013). Improving health information systems for decision making across five sub-Saharan African countries: Implementation strategies from the African Health Initiative. BMC Health Serv Res.

[18] Okello G, Molyneux S, Zakayo S, Gerrets R, Jones C (2019). Producing routine malaria data: an exploration of the micro-practices and processes shaping routine malaria data quality in frontline health facilities in Kenya. Malar J.

[19] Brighton A, D'Arcy R, Kirtley S, Kennedy S (2013). Perceptions of prenatal and obstetric care in Sub-Saharan Africa. Int J Gynecol Obstetr.

[20] Dimagi. CommCare 2021. https://www.dimagi.com/commcare/

[21] Sekhon M, Cartwright M, Francis JJ (2018). Acceptability of health care interventions: a theoretical framework and proposed research agenda. Br J Health Psychol.

[22] Mathole T, Lindmark G, Majoko F, Ahlberg BM (2004). A qualitative study of women's perspectives of antenatal care in a rural area of Zimbabwe. Midwifery.

[23] Uzochukwu B, Onwujekwe O, Akpala C (2004). Community satisfaction with the quality of maternal and child health services in Southeast Nigeria. East Afr Med J.

[24] Grossmann-Kendall F, Filippi V, De Koninck M, Kanhonou L (2001). Giving birth in maternity hospitals in Benin: testimonies of women. Reprod Health Matters.

[25] D'Ambruoso L, Abbey M, Hussein J (2005). Please understand when I cry out in pain: women's accounts of maternity services during labour and delivery in Ghana. BMC Public Health.

[26] Hoyt J, Hill J, Achieng F, Ouma P, Kariuki S, Desai M (2021). Healthcare provider and pregnant women's perspectives on the implementation of intermittent screening and treatment with dihydroartemisinin-piperaquine for malaria in pregnancy in western Kenya: a qualitative study. Malar J.

[27] Hill J, Ouma P, Oluoch S, Bruce J, Kariuki S, Desai M (2020). Intermittent screening and treatment with dihydroartemisinin-piperaquine for the prevention of malaria in pregnancy: implementation feasibility in a routine healthcare system setting in western Kenya. Malar J.

[28] Young N, Taegtmeyer M, Aol G, Bigogo GM, Phillips-Howard PA, Hill J (2018). Integrated point-of-care testing (POCT) of HIV, syphilis, malaria and anaemia in antenatal clinics in western Kenya: a longitudinal implementation study. PLoS ONE.

[29] Winestone LE, Bukusi EA, Cohen CR, Kwaro D, Schmidt NC, Turan JM (2012). Acceptability and feasibility of integration of HIV care services into antenatal clinics in rural Kenya: a qualitative provider interview study. Glob Public Health.

[30] Runge M, Snow RW, Molteni F, Thawer S, Mohamed A, Mandike R (2020). Simulating the council-specific impact of anti-malaria interventions: a tool to support malaria strategic planning in Tanzania. PLoS ONE.

